# Supplementary material for: Artifact Management for Cerebral Near-Infrared Spectroscopy Signals: A Systematic Scoping Review
Source: Bioengineering (Basel). 2024 Sep 18;11(9):933. doi: 10.3390/bioengineering11090933 (PMC11428271; doi:10.3390/bioengineering11090933)
Supplement: Supplementary file 1 [file bioengineering-11-00933-s001.zip › bioengineering-3123326-supplementary.pdf]

Supplementary Materials:

Tobias Bergmann [1,*], Nuray Vakitbilir [1], Alwyn Gomez [2,3], Abrar Islam [1], Kevin Stein [1,4], Amanjyot Singh Sainbhi [1], Logan Froese [5] and Frederick A. Zeiler [1,2,5,6,7,8,*,†]

Table S1. Motion and other disconnection artifact management methods – Accelerometer-based methods

Reference	Subject in-Formation	Data Type (Sampling Rate) – System Used<br><u>Signal measured</u><br>*Auxiliary signals measured*	Sensor location and Instruction to Subjects Signal Recording	Artifact Removal Method(s) Used	Methods Compared	Effectiveness	Study Results and Conclusions	Limitations
Hocke et al. (2023) [49]	10 healthy volunteers, no demographic information provided	fNIRS (6.25 Hz) - ISS Imagent (ISS, Champaign, IL, USA),<br><u>Raw fNIRS converted into HbO and HHb using HOMER2</u><br>*fMRI*<br>*Accelerometer*	Right prefrontal cortex<br>2- and 3-back memory tasks	HighMC with AMARA-fMRI<br>Uses fMRI and acceleration to derive motion traces	UpMC<br>HighMC<br>NoMC	Non-significant channels for HbO in the NoMC and UpMC had no significant difference from HighMC or for HHb<br>Significant channels for HbO in the NoMc and UpMC had no significant difference from HighMC In significant HHb channels, NoMC and UpMC had significant difference from HighMC	Motion artifacts could not be completely resolved with AMARA-fMRI to reveal neuronal activation but had some influence	Limitations on number of artifacts that can be removed when there are very strong artifacts (amount of motion)
Metz et al. (2015) [50]	12 healthy adolescent male volunteers (ages between 10 and 16)	NIRS (35 Hz) - OxyPrem NIRS device<br><u>HbO and HHb</u><br>*Accelerometer (35 Hz)*	Left prefrontal cortex<br>8.5 hours of sleep, two nights (24 recording sessions)	AMARA (MARA + ABAMAR) uses moving standard deviation thresholds in accelerometer to detect artifact (based on acceleration and length of acceleration).	AMARA [47]<br>MARA [41]	Human scorers sensitivity was S = 86% to 96%, ABAMAR S = 92.2%, MARA S = 77.1%, and AMARA = 94.2%<br>** but only when movement detection was considered	ABAMAR had better sensitivity and false positive rate, and time elapsed compared to MARA<br>Most missed artifacts were due to subtle movements	No indications of where data originated<br>Assumption that artifacts are always related to movement

				Spline interpolation with nth order Savitzky-Golay filter<br><br>Reconstruction uses accelerometer data for baseline shift			MARA detected shortest artifacts, AMARA second shortest<br>AMARA not good with non-movement artifacts	Manual parameter values to be chosen<br><br>Only validated against healthy humans
Siddiquee et al. (2018) [36]	4 healthy volunteers (22, 25, 27, and 28 years)	NIRS (1000 Hz) – custom wearable NIRS system based on TI CC3200 chip that incorporates other peripheral chips<br><br><u>HbO and HHb</u><br><br>*3-axis accelerometer, gyroscope, and magnetometer*	N/A<br><br>Two subjects' data contaminated by tapping electrode	Artifacts estimated using autoregressive model with exogenous input (ARX)<br><br>Artifacts identified using inertial measurement unit (IMU) data		In comparison based on the ability of each algorithm to remove artifacts from contaminated data, IMU data with accelerometer and gyroscope and magnometer was much higher in SNR (avg. SNR = 15.38) than accelerometer and gyroscope (avg. SNR = 11.83) as well as the accelerometer only (avg. SNR = 7.35	Better artifact removal using all three IMU signals data, not just accelerometer improved SNR	Small cohort<br><br>Not validated in clinical setting<br><br>Not compared to other methods<br><br>Manually keeping track of time when movement artifacts occurred, should be automated in the future
Sweeney et al. (2010) [51]	1 healthy volunteer, no demographic information provided	NIRS (no sampling rate given) – system used for recording not specified<br><br><u>HbO and HHb</u><br><br>*EEG*<br>*Accelerometer*	Dorsolateral prefrontal cortex<br><br>Three head motions	Two accelerometers (one to detect movement of the subject relative to the position in which the NIRS/EEG is being recorded, and the second to measure movement in electrode or optode)<br><br>Allows for detection of motion artifact and knowing source	Single accelerometer method	Using dual accelerometer data improves quality of signal slightly compared to single accelerometer data	Method can differentiate between subject motion artifacts and sensor displacement artifacts	Single subject<br><br>Not validated in clinical setting<br><br>Not validated with more subjects<br><br>Does not actively remove the artifacts, just flags them

				Reduces time and amount of data needed to be reviewed by technician				
Sweeney et al. (2012) [31]	2 healthy male volunteers, no age demographic information provided	NIRS (25 Hz) - TechEn CW6 (NIRSOptix)<br><br>HbO and HHb<br><br>*Tri-axial accelerometer*	Right prefrontal cortex<br><br>N/A	Adaptive filtering using correlations between motion artifact signals and accelerometer data	Compared against known motion artifact-free signal and accelerometer data	From originally noisy signal the signal to noise ratio raised from -14.37 dB to -8.44 dB<br><br>Improved correlation coefficient for before vs. after adaptive filtering<br><br>Correlation between accelerometer and motion artifact free vs. cleaned signal using adaptive filter were R = 0.777 and R = 0.448, respectively.	Better comparison and performance evaluation of motion artifact removal algorithm by providing good estimate of noise-free signal as reference<br><br>R values indicate the need for a more powerful artifact removal technique	Only applied to portions of data when artifacts were known to be present<br><br>No dramatic improvement in signal quality
Virtanen et al. (2011) [47]	13 healthy volunteers (9 male and 4 female, mean of 26, range of 21 to 32)	NIRS (10 Hz) – frequency domain NIRS device presented by Nissilä et al. [48]<br><br>HHb and HbO<br><br>*Accelerometer*<br>*EEG*<br>*Electrooculography*<br>*Electromyography*<br>*Pulse oximeter*	Single probe on right prefrontal cortex<br><br>N/A	Motion detected by accelerometer based on ABAMAR parameters<br><br>Baseline shift identified in NIRS signal<br><br>Artifact corrected by preceding part of signal if artifact present in both signals	Human panel	ABAMAR agreed with humans on 79% of artifacts and 21% false positive<br><br>Similar performance to human panel	Will not have conflicts like there are in a human panel, lots of disagreement among human panelists<br><br>Can be applied with only a 20 sec delay	Some humans indicated that there were many more artifacts than detected by ABAMAR but no indication as to why this is the case<br><br>ABAMAR ignores motion artifacts when there is no amplitude baseline change. This could be addressed using filtering, but this can make it

								difficult to distinguish between motion related artifacts
Blasi et al. (2010) [42]	24 infants (5 months old)	NIRS (no sampling rate)- two custom NIRS devices compared<br><br><u>HbO</u><br><br>*Accelerometer*	Both temporal lobes<br><br>Exposed to visual stimuli	Reference standard deviation calculated and was used to determine the onset of motion. Thresholding was also conducted simultaneously. Adaptive filter then used to reduce artifacts based on accelerometer motion	Compared performance of two different custom NIRS set ups	6 infants data used with Design 1: ΔSNR = 0.535 dB<br><br>Thresholding removed 16% of data segments that would have passed updated criteria (false negative)	Design 1 is the better design<br><br>Algorithm works better for medium and slow head movement artifact removal	Large number of invalid trials affects algorithms effectiveness<br><br>Only trials that increased SNR were included<br><br>Placement of accelerometer affecting results
Kim et al. (2011) [43]	Number of patients not specified	NIRS (no sampling rate) - custom<br><br><u>HbO and HHb</u><br><br>*Accelerometer with controlled ABP on forearm*	Frontal cortex<br><br>Pressure around arm applied and released, told to exhale and hold breath (creates hypoxic state)	Location of artifacts was estimated using the accelerometer data, adaptive filter removes noise based on accelerometer.	No comparison	Not provided	More so was a presentation of new accelerometer integrated into NIRS device, insufficient information regarding the successfulness in removing different artifacts.	Lag time of 0.5 seconds<br><br>Not tested on more than single patient
von Luhmann et al. (2019) [53]	28 subjects, 16F/12M, 27 right-handed, 28.1 ± 5.8 years	fNIRS (8.33 Hz)<br><br><u>HbO and HHb</u>	Forehead<br><br>Subjects performed n-back tasks	BLISSA²RD involves decomposing fNIRS signals using ICA-ERBM algorithm [52], temporal embedding of accelerometer data,	PCA, Spline and Wavelet-based artifact removal<br><br>ICA-ERBM was compared to fastICA [54]	This algorithm improved the SNR of continuous hemodynamic signals up to 10 dB and reduce motion artifacts by an order of 2, outperforming several conventional	ERBM ICA outperforms fastICA significantly in all metrics ($p \ll 0{:}001$)	Required a minimum number of channels<br><br>Small dataset

	fNIRS-EEG device, added accelerometer (50 Hz)	identification of shared components using CCA, and estimation of artifacts in ERBM source space before providing cleaned signal		methods in extracting the HRF<br>In HbO correlation and RMSE, the BLISSA^{2}RD algorithm had superior performance to all other methods<br><br>In HHb correlation, spline outperformed BLISSA^{2}RD algorithm; however, BLISSA^{2}RD outperformed both spline and PCA significantly in RMSE (no significant improvement compared to wavelet-based method)	BLISSA^{2}RD performs well in HbO, and decently well in HHb.

Where ABAMAR= Accelerometer-Based Motion Artifact Removal, ARX = Autoregressive Model with Exogenous Input, BLISSA^{2}RD= Blind Source Separation and Accelerometer based Artifact Rejection and Detection, CCA= Canonical Correlation Analysis, EEG= Electroencephalography, EMG= Electromyography, EOG= Electrooculography, ERBM= Entropy Rate Bound Minimization, fMRI=functional magnetic resonance imaging, fNIRS=Functional Near-Infrared Spectroscopy, HighMC= High Sampling Rate Motion Correction, HHb= Concentration of Deoxyhemoglobin, HbO= Concentration of Oxyhemoglobin, ICA= Independent Component Analysis, IMU=Inertial Measurement Unit, MARA=Movement Artifact Reduction Algorithm, NIRS=Near-Infrared Spectroscopy, N/A=Not Applicable, PCA= Principal Component Analysis, SNR= Signal-to-Noise Ratio

Table S2. Motion and other disconnection artifact management methods – Wavelet-based methods.

Reference	Subject Information	Data Type (sampling rate) – System Used<br><br>Signal Measured<br><br>*Auxiliary Signals measured*	Sensor Location and Instruction to Subjects Signal Recording	Artifact Removal Method(s) Used	Methods Compared	Effectiveness	Study Results and Conclusions	Limitations
Bergmann et al. (2023) [55]	83 TBI (57 male), Age: 42 (27.5–58.6)<br><br>103 healthy volunteer (68 male)<br>Age: 26 (22–31)<br><br>27 SP (22 male)<br>Age: 57 (52–65.5)	NIRS (1 Hz) - Covidien INVOS 5100C or 7100<br>rSO_2<br><br>*ABP*	Left and right prefrontal cortex<br><br>N/A	Continuous Morlet wavelet transformation using coherence and coefficient thresholds to identify artifacts	N/A	The removal success rates in HC, SP, and TBI populations were 100%, 99.8%, and 99.7%, respectively (though it had limited precision in determining the exact point in time)	Method described able to remove signal loss artifacts<br><br>No better performance than typical thresholding	Sampling rate of NIRS technology was only 1 Hz and as such, cardiac frequency bands not able to be identified
Chiarelli et al. (2015) [56]	20 healthy volunteer with mean age of 42	fNIRS (10 Hz) - frequency-domain NIR spectrometer (ISS Imagent, Champaign, Illinois)<br><br>Optical density signal	Optodes scattered across entire scalp surface<br><br>Subjects told to think about nothing	kbWF is discrete wavelet transformation method	Compared to different DWF in how artifact occurring is decided, Principle Component Analysis (PCA), targeted PCA (tPCA), and spline interpolation	kbWF algorithm showed largest improvements in MSE (decrease of 24%) and SNR (increase of 55%)<br><br>Wavelet filtering performed decent with MSE (decrease of 7%) and SNR (increase of 42%)	kbWF yielded lower MSE and better SNR<br><br>These data indicate that application of the kbWF algorithm results in a reduction of MSE in 95% of the cases, and in an increase of MSE in only 5% of the cases (and even in these cases the increase is very small).<br><br>All others motion correction algorithms performed at a lower level, showing	Inability to correct for lasting shifts in light intensity<br><br>Shifts would not be detected by kbWF because they would affect first or second level of DWT, can be overcome with high pass for

							improvements in a number of cases varying between 12% (for PCA) and 71% (for F)	longer recording periods
Hossain et al. (2022) [57]	10 subjects from PhysioNet databank, assumed healthy volunteer, no demographic informationn provided<br><br>16 data sets	fNIRS (25 Hz) – signal type or recording hardware not explicitly stated<br><br>Likely HbO and HHb (corrupted and ground truth signals)	Measured on scalp<br><br>N/A	WPD is used to decompose signal at varying scales using different wavelet packages (Daubechies, Symlets, Coiflets, Fejer-Korovkin)<br><br>Canonical Correlation Analysis (CCA) is used to dissociate mixed and noisy signals<br><br>Polynomial curve fitting to estimate baseline in cases of baseline drift	Comparison between each wavelet package with CCA (two-stage) and without CCA (one-stage)	Best performance was by WPD_{fk4} with highest reduction in artifacts (26.40%) and greatest change in SNR (16.11 dB) of all single stage motion artifact correction techniques<br><br>Best performance in two-stage for SNR was WPD_{db1}-CCA with change in SNR of 16.55 dB, and best for avg. motion artifact removal was WPD_{fk8}-CCA with 41.40%	Efficacy measured using signal-to-noise ratio (SNR) and percentage reduction in artifacts (metric based on clean signal)<br><br>Varying wavelet decomposition parameters (some including CCA) were evaluated<br><br>Best performance in two-stage for SNR was WPD_{db1}-CCA and best for avg. motion artifact removal was WPD_{fk8}- CCA	WPD-CCA technique is not be able to identify the motion corrupted<br><br>CCA components in the without a ground truth signal, may need some kind of autocorrelation function
Molavi and Dumont (2010) [22]	2 healthy volunteer newborn children (1 and 2 days old)	fNIRS (10 Hz) – Hitachi ETG-4000<br><br>Raw optical density data converted to chromophore concentration changes	Right and left temporal lobes<br><br>N/A	TIWT is discrete wavelet transformation (DWT) method with shift operator	Median filter<br><br>Discrete wavelet transformation method SURE threshold	The mean artifact power attenuation between the two subjects was -17.26 dB and the average NMSE was -13.99 dB	TIWT yields overall better performance, demonstrated in clinical setting	Needs further validation on more subjects. Could not be compared to some other methods due to only having NIRS recorded

Molavi and Dumont (2012) [23]	3 healthy volunteer infants (1 male) that are two days old (1) and one day old (2)	NIRS (10 Hz) - Hitachi ETG-4000<br><br><u>Raw optical density data</u>	Left and right temporal regions<br><br>N/A	Discrete wavelet transformation (Daubechies) used to detect abrupt changes in NIRS signals	Adaptive denoising method	Artifact attenuation is much greater in proposed method than adaptive wavelet denoising method (SURE method) using NMSE (−13.80, −17.54 and −14.84 dB for each of the three subjects)<br><br>No quantification for comparison with other methods included in article	Method is good for motion artifacts<br><br>Algorithm functions better for large spike-like artifacts	Small cohort<br><br>Poor depiction of success of algorithm at removing artifacts<br><br>Variance in artifact attenuation between subject datasets could be due to artifact type (ex. spike-like artifacts with high amplitudes are easier for DWT to detect)<br>Method cannot be used in real-time processing
Perpetuini et al. (2021) [58]	25.5 ± 8.5 years<br><br>16 healthy volunteer (9 males/7 females)	fNIRS (10 Hz)- Octamon fNIRS device (Artinis Medical Systems)<br><br><u>Optical density</u><br><br>*IRT (10 Hz) with tracking using 2-D cross-correlation between a target template (TT)*	Frontal cortex<br><br>Asked to perform certain controlled head motions for portions of recording	CWT Morse wavelet analysis using coherence and coefficients<br><br>Video tracking of motion of detector to obtain motion vector<br><br>Reconstruct signal using inverse wavelet transform	Other wavelet analysis<br><br>PCA- based<br><br>Spline-based<br><br>Correlation-based	Largest SNR was for threshold of 0.6 for CWT Morse wavelet coefficients and coherence<br><br>CWT Morse wavelet method had highest SNR (close to 5.5), lowest MSE (below 1), highest beta values (close to 0.9), highest t-stat (close to 22)	Good performances in both IR tracking and of motion correction<br><br>Proposed method increases the capabilities of the general linear model-based methods to assess cortical activation, improving statistical analysis of fNIRS	May be difficult to extend monitoring in entire head (not just frontal cortex) due to frame size of IRT camera<br><br>Not applicable in outdoor locations

Wei et al. (2018) [59]	40 healthy volunteer 16 male (mean age is 32)	NIRS (10 Hz) – no equipment information provided<br><br><u>HbO</u><br><br>*3D acceleration (10 Hz)*	Prefrontal cortex<br><br>Sleeping	Discrete wavelet transformation, dual thresholds	Compared to wavelet filtering [23]	The proposed method had a SNR value above 0 dB and an R^2 value above 0.4. Wavelet filtering had an SNR value below -10 dB and R^2 value close to 0.	Strong removal of high-frequency spike artifacts, poorer in baseline shifts	When baseline shifts occur frequently (e.g. <50 s) the performance of the algorithm will be reduced.<br><br>Assumes artifacts last for a short time
Robertson et al. (2010) [17]	3 subjects, demographic information not specified	NIRS (1.8 Hz) – NIRX DYNOT<br><br>Used wavelengths corresponding to HbO and HHb, but did not state	Left and right motor cortices<br><br>Shake, tilt, and nod head (3 subjects), finger tapping (1 subject)	Two-input Recursive Least Squares Adaptive Filter<br><br>**Discrete wavelet (symlet 8 wavelet) transformation using thresholding of coefficients**<br><br>Two channel multiple regression<br><br>Multi-channel (30) regression<br><br>ICA-based method	Each methodology presented in article was compared to each other	When time of motion was known (set of 3 subjects) the average across the three subjects for SNR (dB):<br>For λ=760nm, Wavelet (5.95), 30-channel regression (5.67) and ICA (5.62) had the best performance. For λ=830nm, the ranking was the same but Wavelet (4.93) and 30-channel regression (4.56) performed dramatically better than ICA (2.75) in this occasion.<br><br>However, when the motion was not known: ICA (λ=760nm - SNR= 3.20 dB; λ=830nm SNR= 3.67 dB) and 30-channel regression (λ=760nm - SNR= 3.01 dB; λ=830nm - SNR= 2.54 dB) outperformed wavelet (λ = 760nm – SNR = 0.89 dB; λ = 830nm – SNR = 0.58 dB).<br><br>The RLS method and 2-channel regression method had	ICA and 30-channel regression performed the best<br><br>Wavelet performs well when the location of the motion is known	Multi-channel regression and ICA requires the use of several channels<br><br>Without known motion, the SNR when wavelet is used drops dramatically<br><br>Small cohort

						substantially worse performance than the rest.		

Where CCA = Canonical Correlation Analysis, dbN= Debauchies (wavelet type), DWF=Discrete Wavelet Function, fNIRS=Functional Near-Infrared Spectroscopy, fkN=Fejer-Korovkin (wavelet type), HHb=Concentration of Deoxyhemoglobin, HbO=Concentration of Oxyhemoglobin, kbWF=Kurtosis-Based Discrete Wavelet Transformation Method, MSE=Mean Squared Error, N/A=Not Applicable, NIRS=Near-Infrared Spectroscopy, NoMC= No Motion Correction, NMSE=Normalized Mean Squared Error, PCA=Principle Component Analysis, R^2=Coefficient of Determination, rSO_2=Cerebral Regional Oxygen Saturation, SNR=Signal-to-Noise Ratio, SP=Elective Spinal Surgery Patients, SURE=Stein's Unbiased Risk Estimator, TBI=Traumatic Brain Injury Patients, TIWT=Translation Invariant Wavelet Transform, tPCA=Targeted Principle Component Analysis, UpMC= Up Sampled Motion Correction, WPD=Wavelet Packet Decomposition

Table S3. Motion and other disconnection artifact management methods – Machine learning-based methods.

Reference	Subject Information	Data Type (sampling rate) – System Used<br><br>Signal Measured<br><br>*Auxiliary Signals Measured*	Sensor Location and Instruction to Subjects Signal Recording	Artifact Removal Method(s) Used	Methods Compared	Effectiveness	Study Results and Conclusions	Limitations
Russell-Buckland et al. (2018) [37]	8 healthy volunteers, demographic information not provided	Broadband NIRS – custom equipment<br><br>No signal type provided	N/A<br><br>6 artifacts simulated, some movement based (head movement)and some induced using ambient light	Feature engineering used to try to identify artifacts. Features chosen: power density fraction, sample entropy, autocorrelation, and area under the curve. Data is then fed into Random Forest Algorithm Machine learning algorithm<br><br>Uses 6 data sets to train, 2 to test	N/A	Long-separation sensor was better in scoring for light only scores by weighted F1-score metric.	Algorithm struggled in general to identify movement artifacts	Engineering features of algorithm or not enough information<br><br>Algorithm shows difficulty identifying motion artifacts. May need to use accelerometers and external light sensors as opposed to

								broadband NIRS
Kim et al. (2022) [62]	42 healthy subjects, 20F/22M (ages 22 to 40)	fNIRS (10 Hz) – Artinis OxyMon<br><br>HHb and HbO	Prefrontal areas of both hemispheres<br><br>Cycle through instructions, action (moving body), rest	DL architecture extracts features from fNIRS data by differentiating motion artifacts from HRF. Model will reduce motion artifacts by reconstructing HRF<br><br>21 training (set 1) and 21 testing (set 2) split for datasets	Compared to:<br>AR-based model [60]<br><br>Wavelet-MDL method (WV1) [61]<br><br>Hybrid method between AR and wavelet-MDL (WV2)	The metrics used for comparison included the MSE and performance of activation detection measured using AUC-ROC<br>For short tasks: MSE for DL-based method was the smallest, for training set, DL, WV, and AR, had similar AUC-ROC. DL performed better for set 2<br>For long task**:** MSE was generally higher, but DL had the best performance (difference between MSE had more statistical significance). Detection efficiency decreased in AUC-ROC for long tasks; however, DL perofmed the best	Proposed method is able to extract HRF more effectively than the wavelet-based and autoregressive based models<br><br>This was conducted for a variety of different artifacts to demonstrate its efficacy.	Dataset was based off real artifacts; however, synthetic HRF was added<br><br>Established only for a single fNIRS system
Lee et al. (2018) [64]	6 subjects, two stroke patients and four healthy (34 to 57 years)	fNIRS (10 Hz) – FOIRE-3000<br><br>HbO	Covering sensory and motor areas of cortex<br><br>Walking on ground or walking on treadmill	Motion artifact detection from contaminated optode using signal entropy imbalance<br><br>10-band wavelet transformation conducted and fed into back propagation neural network (BPNN) with AdaM optimizer [63] trained using MSE function and uses multiple linear regression	HRF smoothing method [65]<br><br>Wavelet denoising<br><br>Wavelet-MDL [61]	CNR calculated for each subject for both corrected channels and channels in regions of interest that corresponded to the walking task conducted by the subjects:<br><br>Success of methods in corrected channels, ROI channels:<br>1. Proposed method (0.63,0.73)<br>2. HRF smoothing (0.49, 0.74)<br>3. Wavelet denoising (0.36, 0.64)	Wavelet MDL had poor performance<br><br>Wavelet denoising improved CNR but not by much<br><br>HRF smoothing performs well in ROI signals but when data has low CNR, performance falters<br><br>Proposed method can be used for global detrending	May have performance hindered if the algorithm is applied to highly artifact ridden datasets<br><br>Need to improve the artifact ridden data segment detection

						4. Wavelet MDL (-0.12, 0.09)		

Where AR= Autoregressive, AUC-ROC= Area Under Receiver Operator Characteristic Curve, BPNN= Back Propagation Neural Network, CNR= Contrast to Noise Ratio, DL= Deep Learning, HbO= Concentration of Oxyhemoglobin, HHb= Concentration of Deoxyhemoglobin, HRF= Hemodynamic Response Function, MDL= Minimum Description Length, MSE= Mean Squared Error, N/A=Not Applicable, NIRS=Near-Infrared Spectroscopy, ROI= Region of Interest, WV= Wavelet

Table S4. Motion and other disconnection artifact management methods – Filter-based methods.

Reference	Subject Information	Data Type (Sampling Rate) – System Used<br><br>Signal Measured<br><br>*Auxiliary signals measured*	Sensor location and Instruction to Subjects Signal re-Cording	Artifact Removal Method(s) Used	Methods Compared	Effectiveness	Study Results and Conclusions	Limitations
Izzetoglu et al. (2005) [38]	11 healthy volunteers, no demographic information provided	NIRS (1.6 Hz) – custom 16-channel recording system<br><br>Signal type not explicitly stated, likely HbO and HHb<br><br>*Accelerometer*	Optodes on dorsolateral prefrontal cortex<br><br>Performing three specific head movements	Wiener filtering used to develop linear time invariant filter, accelerometer used to detect incidence of artifact	Adaptive filter	Average change in correlation coefficients for Wiener and adaptive filters are 0.3438 and 0.09913, respectively.<br><br>Average change in signal to noise ratio for Wiener and adaptive filters are 6.6879 and 3.4396, respectively.	Improvements in both SNR and correlation coefficients in proposed Wiener method<br><br>Superior performance in Wiener method	Requires further improvements to be used for online data as it currently needs all data before algorithm can execute<br><br>Only tested on head movements of a specific type
Izzetoglu et al. (2010) [39]	11 healthy volunteers, no demographic information provided	NIRS (1.6 Hz) – custom 16-channel recording system<br><br>Signal type not explicitly stated, likely HbO and HHb	Pre-frontal cortex<br><br>Performing three specific head movements (slow, medium, and	Kalman filtering method that uses autoregressive state space representation and least squares estimation method for estimating signals that can be buried within noise	Adaptive filter<br><br>Wiener filter	Comparison of Adaptive, Wiener, and Kalman filters for slow, medium, and fast head movements yielded average change SNR for each filter as 3.4396, 6.6980, and 7.6548, respectively	Proposed method is significantly better in SNR than adaptive filter<br><br>No significant improvement for Kalman compared to Wiener filter	Kalman filter can have build-up of errors as prediction time increases in Kalman filtering, non-linearity in the system itself. This can be

			fast motions)					mitigated through backward Kalman smoother, but this must be applied offline after all data has been collected (cannot be applied in real-time)
Amian and Setarehdan (2013) [44]	6 healthy right handed subjects (3 males)	fNIRS (no sampling rate) – custom NIRS system<br><br><u>HHb and HbO</u>	Forehead<br><br>Instructions included sitting still, moving head around	Extends the Izzetoglu method that uses auto-regression model to translate the signal into the state space to apply Kalman filter<br><br>This method uses an ARMA model instead to initially model the motionless signal and then uses a Kalman filter.	Compared to auto-regressive (AR) method	ΔSNR for AR with Kalman = 8.7 dB<br><br>ΔSNR for ARMA = 10.4 dB	ARMA includes more terms than AR, as such can be better fitted to different systems<br><br>Both AR and ARMA can estimate white Gaussian noise<br><br>ARMA is good at estimating motionless signal from motion artifact ridden signal	Relies upon time where patient is motionless
Huang et al. (2022) [66]	8 subjects 2F/6M, (26.9 ± 2.75 years)	fNIRS (52.08 Hz) – ISS Imagent<br><br><u>HbO and HHb</u>	Frontal cortex	Dual-stage median filter method to remove spike artifacts and “step like” artifacts, window sizes for each filters.	Spline interpolation [41]<br><br>Wavelet-based method [23]	Better performance by SDR and NMSE in both wavelengths evaluated with averages of 1.185 and 0.63, respectively.	DSMF has simple structure<br><br>Able to remove low frequency drift, using simulated data<br><br>No experiential knowledge required	When both types of artifacts are close together, this algorithm struggles<br><br>Seems like this study would have had more significance if a larger number of subjects had been used, merely proof of

								concept at present.
Dong and Jeong (2019) [45]	23 subjects (demographic information not provided)<br><br>Data was collected in past article from same authors [73]	fNIRS (50 Hz)-custom built system<br><br><u>HbO and HHb</u><br><br>Recorded and converted into a synthetic signal	Prefrontal cortex<br><br>Short and long separation conducted for recording	Pre-processing to detect motion artifacts using two-sided standard deviation<br><br>Extended Kalman filter (EKF)	LKF<br><br>AF [72]	In segments with motion artifacts using short separation: RMSE and PRD reduced by more than 40% in HbO and HHb signals using EKF compared to LKF. Average of improvements of 3 evaluation parameters (RMSE, PRD, correlation coefficient) between recovered and true HRF had 34% increase in HbO and 62% in HHb compared to LKF<br><br>By metrics of RMSE, PRD, and correlation coefficient, ranking of successfulness was:<br>1) EKF, 2) LKF 3) AF regardless of signal type (HbO or HHb) and if there were motion artifact present	EKF has lower RMSE and PRD as well as higher correlation compared to LKF and AF regardless of the presence of motion artifacts.<br><br>EKF reduced noise in HHb more than HbO	Synthetic data that is based off of true motion artifacts detected but is based off of data segments classified as having or not having artifacts with a synthetic HRF added
Robertson et al. (2010) [17]	3 subjects, demographic information not specified	NIRS (1.8 Hz) – NIRX DYNOT<br><br>Used wavelengths corresponding to HbO and HHb, but did not state	Left and right motor cortices<br><br>Shake, tilt, and nod head (3 subjects), finger tapping (1 subject)	**Two-input Recursive Least Squares Adaptive Filter**<br><br>Discrete wavelet (symlet 8 wavelet) transformation using thresholding of coefficients<br><br>Two channel multiple regression	Each methodology presented in article was compared to each other	When time of motion was known (set of 3 subjects) the average across the three subjects for SNR (dB):<br>For λ=760nm, Wavelet (5.95), 30-channel regression (5.67) and ICA (5.62) had the best performance. For λ=830nm, the ranking was the same but Wavelet (4.93) and 30-channel regression (4.56) performed dramatically better	ICA and 30-channel regression performed the best<br><br>Wavelet performs well when the location of the motion is known	Multi-channel regression and ICA requires the use of several channels<br><br>Without known motion, the SNR when wavelet is used drops dramatically<br><br>Small cohort

				Multi-channel (30) regression<br><br>ICA-based method		than ICA (2.75) in this occasion.<br><br>However, when the motion was not known: ICA (λ = 760nm – SNR = 3.20 dB; λ = 830nm SNR = 3.67 dB) and 30-channel regression (λ = 760nm – SNR = 3.01 dB; λ = 830nm – SNR = 2.54 dB) outperformed wavelet (λ = 760nm - SNR= 0.89 dB; λ = 830nm – SNR = 0.58 dB).<br><br>The RLS method and 2-channel regression method had substantially worse performance than the rest.		

Where AF= Adaptive Filtering, EKF= Extended Kalman Filter, HHb= Concentration of Deoxyhemoglobin, HbO= Concentration of Oxyhemoglobin, LKF= Linear Kalman Filter, NIRS=Near-Infrared Spectroscopy, NMSE= Normalized Mean Squared Error, PRD= Percent Root Difference, SNR=Signal-to-Noise Ratio, RMSE= Root Mean Squared Error, SDR= Signal Distortion Ratio. **Bold text indicates that there were several methods that were presented in the article, bold indicates the highlighted method.**

Table S5. Motion and other disconnection artifact management methods – Component analysis-based methods.

**Reference**	**Subject Information**	**Data Type (sampling rate) – System Used**<br><br>**Signal Measured**<br><br>***Auxiliary Signals Measured***	**Sensor location and Instruction to Subjects Signal Recording**	**Artifact Removal Method(s) Used**	**Methods Compared**	**Effectiveness**	**Study Results and Conclusions**	**Limitations**
Yanhua Shi et al. (2015) [67]	4 healthy volunteers (3 male) with mean age of 23 years	fNIRS (55.56 Hz) – LABNIRS system (SHIMAOZU)	Optodes placed on motor left cortices	ICA decomposes linear mixture of signals into statistically independent source signals	Wavelet-based method	Mean SNR improved for Hb and HbO using proposed method by 2.336 and 2.139, respectively. Compared to wavelet method with Hb and	Superior performance compared to a wavelet-based method	Small cohort<br><br>Will performance be hindered if used

		HbO and HHb	Subjects asked to move their heads in specific movements	Independent components used to mark channels with artifacts (use the notion that Hb and HbO signals caused by motion are positively correlated)		HbO increasing 1.191 and 1.118	Evaluated using signal to noise ratio<br><br>Mean SNR better for ICA than wavelet method	with lower sampling rate data
Yücel et al. (2014) [28]	5 healthy volunteers (4 male) 23-52 years old	NIRS, typical sampling rate of equipment used was 25 Hz – TechEn CW6 (NIRSOptix)<br><br>HbO and HHb	NIRS data from collodion-fixed optical fibers (left motor region) and Velcro-based probe (right motor region)<br><br>5 actions were performed	Typical PCA applies orthogonal transformation, targeted PCA applies procedure to parts of signal with known motion artifacts<br><br>Artifacts identified using thresholding	Spline method<br><br>Wavelet method	Compared between raw data HRF after applying method and to true HRF. tPCA had best improvement in MSE compared to wavelet, worse than spline interpolation. tPCA showed highest correlation with true HRF and had best Pearson correlation value tPCA improves largest improvement of MSE and R^2. MSE obtained for tPCA was lower than wavelet-based correction and no correction. tPCA and spline interpolation produce results closer to the true HRF. Wavelet-based filtering captures the shape of HRF well but is lower amplitude.	HRF recovered with the collodion-fixed fiber probe, which is better for motion artifacts, is much better even without correction<br><br>PCA for motion correction is much better for HRF recovery compared to spline and wavelet-based correction methods<br><br>Spline and tPCA correct for baseline shifts near motion artifact epoch<br><br>Wavelet filtering performs well with Velcro side, it increases the MSE for the collodion-fixed probe	Small cohort<br><br>No presentation of sampling rate used<br><br>tPCA method would not perform as well in a situation where cerebral activation and motion artifacts are correlated
von Luhmann et al. (2019) [53]	28 subjects, 16F/12M, 27 right-handed, 28.1 ± 5.8 years	fNIRS (8.33 Hz)<br><br>HbO and HHb<br><br>*fNIRS-EEG device, added accelerometer (50 Hz)*	Forehead<br><br>Subjects performed n-back tasks	BLISSA²RD involves decomposing fNIRS signals using ICA-ERBM algorithm [52], temporal embedding of accelerometer data, identification of shared components using CCA, and estimation of artifacts in ERBM source space	PCA, Spline and Wavelet-based artifact removal<br><br>ICA-ERBM was compared to fastICA [54]	This algorithm improved the SNR of continuous hemodynamic signals up to 10 dB and reduce motion artifacts by an order of 2, outperforming several conventional methods in extracting<br><br>In HbO correlation and RMSE, the BLISSA²RD	ERBM ICA outperforms fastICA significantly in all metrics ($p \ll 0{:}001$)<br><br>BLISSA²RD performs well in HbO, and decently well in HHb.	Required a minimum number of channels<br><br>Small dataset

				before providing cleaned signal		algorithm had superior performance to all other methods<br><br>In HHb correlation, spline outperformed BLISSA²RD algorithm; however, BLISSA²RD outperformed both spline and PCA significantly in RMSE (no significant improvement compared to wavelet-based method)		
Robertson et al. (2010) [17]	3 subjects, demographic information not specified	NIRS (1.8 Hz) – NIRX DYNOT<br><br>Used wavelengths corresponding to HbO and HHb, but did not state	Left and right motor cortices<br><br>Shake, tilt, and nod head (3 subjects), finger tapping (1 subject)	Two-input Recursive Least Squares Adaptive Filter<br><br>Discrete wavelet (symlet 8 wavelet) transformation using thresholding of coefficients<br><br>Two channel multiple regression<br><br>Multi-channel (30) regression<br><br>**ICA-based method**	Each methodology presented in article was compared to each other	When time of motion was known (set of 3 subjects) the average across the three subjects for SNR (dB):<br>For λ = 760nm, Wavelet (5.95), 30-channel regression (5.67) and ICA (5.62) had the best performance. For λ = 830nm, the ranking was the same but Wavelet (4.93) and 30-channel regression (4.56) performed dramatically better than ICA (2.75) in this occasion.<br><br>However, when the motion was not known: ICA (λ = 760nm – SNR = 3.20 dB; λ = 830nm SNR = 3.67 dB) and 30-channel regression (λ = 760nm – SNR = 3.01 dB; λ = 830nm – SNR = 2.54 dB) outperformed wavelet (λ = 760nm - SNR= 0.89 dB; λ = 830nm – SNR = 0.58 dB).	ICA and 30-channel regression performed the best<br><br>Wavelet performs well when the location of the motion is known	Multi-channel regression and ICA requires the use of several channels<br><br>Without known motion, the SNR when wavelet is used drops dramatically<br><br>Small cohort

						The RLS method and 2-channel regression method had substantially worse performance than the rest.		

Where BLISSA²RD= Blind Source Separation and Accelerometer based Artifact Rejection and Detection, CCA= Canonical Correlation Analysis, ERBM= Entropy Rate Bound Minimization, fNIRS=Functional Near-Infrared Spectroscopy, HHb= Concentration of Deoxyhemoglobin, HbO= Concentration of Oxyhemoglobin, ICA=Independent Component Analysis, MSE=Mean Squared Error, N/A=Not Applicable, NIRS=Near-Infrared Spectroscopy, HRF=Hemodynamic Response Function, PCA=Principal Component Analysis, R^2=coefficient of determination, RMSE= Root Mean Squared Error, SNR=Signal-to-Noise Ratio, tPCA=Targeted Principle Component Analysis, WPD=Wavelet Packet Decomposition. **Bold text indicates that there were several methods that were presented in the article, bold indicates the highlighted method.**

Table S6. Motion and other disconnection artifact management methods – Hybrid methods.

**Reference**	**Subject Information**	**Data Type (Sampling Rate) – System Used**<br><br>**<u>Signal Measured</u>**<br><br>***Auxiliary Signals Measured***	**Sensor Location and Instruction to Subjects Signal Recording**	**Artifact Removal Method(s) Used**	**Methods Compared**	**Effectiveness**	**Study Results and Conclusions**	**Limitations**
Gao et al. (2022) [40]	40 healthy volunteers (16 male and 24 females, mean age of 32)	fNIRS (10 Hz) – custom equipment<br><br><u>HbO and HHb</u>	Left and right prefrontal cortex<br><br>N/A	Hybrid method uses:<br><br>Dynamic thresholding<br><br>Large oscillations: cubic spline interpolation<br><br>Baseline shift correction: spline interpolation<br><br>Slight oscillation correction: dual threshold wavelet-based method	Wavelet-based [23]<br><br>Accelerometer-based [47]<br><br>Spline interpolation [41]<br><br>Median filtering<br><br>Spline Savitzky-Golay [32]<br><br>Spline-Rloess<br><br>Severe oscillation processed with cubic spline interpolation combined with wavelet filtering	For the hybrid method proposed the R value between the process and clean signal was close to 0.8 (none of the other methods are above 0.6) and the SNR between these two signals was above 0 for the hybrid method (none of the other methods were above 0)	Proposed method indicated superior performance compared to improvement methods such as wavelet-based, accelerometer-based, spline interpolation, median filtering, spline Savitzky-Golay, spline-Rloess, and severe oscillation processed with cubic spline interpolation combined with wavelet filtering	Challenges in removing noises and artifact induced by fNIRS measurements due to how erratic they are in origin and form

Jahani et al. (2018) [32]	Set #1 - 7 healthy volunteers<br><br>Set #2 – 5 healthy volunteers<br><br>No demographic information provided	fNIRS (50 Hz) – TechEn CW6 (NIRSOptix)<br><br>HbO and HHb	Covering most of prefrontal cortex<br><br>Set #1 - performing prescribed actions<br><br>Set #2 – at rest	Combined spline interpolation and Savitzky-Golay (SG) filtering for artifact/ baseline shift removal and data smoothing, respectively	Wavelet filtering [23]<br><br>Spline-Rloess<br><br>tPCA [28]<br><br>CBSI [24]	Each method was compared to the true HRF by metrics of mean-squared error (MSE), peak-to-peak error (Ep), coefficient of determination (R^2), and area under the receiver operator characteristic curve (AUC-ROC). For the first data set, a combined method of Rloess and spline interpolation (Rloess-spline) had the best performance in metrics MSE (0.60 ± 0.16 x 104), Ep (3.90 ± 1.13 x 104), R^2 (0.80 ± 0.02), and CSBI had the best performance in AUC-ROC (0.91 ± 0.03). For the second data set, the proposed spline-SG method had the best performance in metrics MSE (0.44 ± 0.06 x 104) and Ep (2.52 ± 0.41 x 104), CSBI had the best performance in R2 (0.84 ± 0.01). and a spline only method, the proposed spline-SG, and spline-Rloess method had identical AUC-ROC results (0.89 ± 0.05).	Results indicate that spline-SG method deals well with baseline shifts as well as high-frequency spikes, as a result, SG does not have to deal with baseline shifts (as it is dealt with by spline), also does not have long processing time	
Scholkmann et al. (2010) [41]	3 datasets used, demographic information not provided	NIRI (sampling rate not presented) – custom equipment<br><br>HbO, HHb, and tHb (Three types of artifacts: short impulses, baseline shifts, temporally limited low	N/A<br><br>Subjects performed finger tapping exercise	Movement artifact reduction algorithm (MARA) includes 6 steps using moving standard deviation (detect motion artifacts) and spline interpolation (replace artifacts)	N/A	Average percental change in PRD, RSME, and R were 89.7% decrease, 89.8% decrease, and 61.6% increase	Validated with real and simulated NIRI data<br><br>Improvement in signal quality ion both synthetic and real NIRI data	Small cohort<br><br>No subject information<br><br>No quantification of improvement with real signal, only simulated

		frequency oscillations)						
Zhou et al. (2021) [46]	4 subjects, demographic information not included	fNIRS (sampling rate not presented) – custom equipment<br><br><u>HbO and HHb</u>	Frontal lobe<br><br>Subjects told to shake head, nod, move mouth, frown, and move eyes<br><br>Contaminated dataset' had motion artifacts added	Movement artifact removal algorithm uses a moving standard deviation (MSD) to detect the onset of artifacts in signal (either drift or spikes). Cubic spine interpolation is then used for fitting the result to remove the artifact. The MSD filter is then shrunk to detect smaller and SG filtering is used to denoise the signal.<br><br>Classification of tasks in combination with several machine learning classification algorithms including SVM, DT, KNN and Random Forest	Only spline interpolation<br><br>Only SG filtering	SNR used to quantify effectiveness:<br><br>Spline: -19.96 dB<br>SG: -23.79 dB<br>Proposed: 2.41 dB<br><br>Deep forest algorithm had the best performance in prediction accuracy on raw data set, contaminated data set, and corrected data set with prediction accuracies of 87.10%, 74.73%, and 83.70%, respectively.	Proposed algorithm was successful in correcting the dataset by removing artifacts<br><br>Deep forest had the best performance in classification of artifacts on all datasets.	Small cohort
Gu et al. (2016) [27]	13 children (ages 6 to 9)	fNIRS (10 Hz) – Hitachi ETG-4000<br><br><u>HbO</u>	Frontal cortex and temporal cortex<br><br>Subjects asked not to move	Proposed EMD-based method has five steps. Algorithm involves motion detection using thresholding, EMD into IMFs, artifact removal using correlation between IMF and original data, and using spline interpolation to maintain continuity.	Spline interpolation [41]<br><br>Wavelet filtering [23]<br><br>Kurtosis based wavelet filtering [56]	Average increase in SNR:<br>1. EMD (53%)<br>2. SI (51%)<br>3. WF (35%)<br>4. kbWF (23%)<br><br>Average MSE reduction across channels:<br>1. WF (59%)<br>2. EMD (47%)<br>3. kbWF (43%)<br>3. SI (42%)	EMD method had good performance in R^2 and second best performance in SNR and MSE.<br><br>Overall EMD was the best method, good for non-stationary data and correct baseline shifts<br><br>kbWF does not perform well with all motion artifacts	Dependent on detection of motion artifacts being accurate.

				Calculation of R^2 of output from method compared to 'true signal'. All methods performed better than no correction, EMD performed the best (R^2 = 0.79)	WF only good with spike artifacts	

Where AUC-ROC=Area Under Receiver Operating Characteristic Curve, CBSI=Correlation Based Signal Improvement, EMD= Empirical Mode Decomposition, E_p=Peak-to-Peak Error, fNIRS=Functional Near-Infrared Spectroscopy, HHb= Concentration of Deoxyhemoglobin, HbO= Concentration of Oxyhemoglobin, HRF=Hemodynamic Response Function, IMF= Intrinsic Mode Functions, kbWF= Kurtosis-based Wavelet Filtering, MARA=Movement Artifact Reduction Algorithm, MSE=Mean Squared Error, MSD= Moving Standard Deviation, N/A=Not Applicable, NIRI=Near-Infrared Imaging, NIRS=Near-Infrared Spectroscopy, PRD=Percent Root Difference, R=Pearson's Correlation Coefficient, R^2=Coefficient of Determination, RMSE=Root Mean Squared Error, SG=Savitzky-Golay Filtering, SI= Spline Interpolation, SNR=Signal-to-Noise Ratio, WF= Wavelet Filtering

Table S7. Motion and other disconnection artifact management methods – Other methods.

Reference	Subject Information	Data Type (sampling rate) – System Used<br><br><u>Signal Measured</u><br><br>*Auxiliary Signals Measured*	Sensor Location and Instruction to Subjects Signal Recording	Artifact Removal Method(s) Used	Methods Compared	Effectiveness	Study Results and Conclusions	Limitations
Barker et al. (2013) [60]	22 healthy volunteer children (3 to 5 years old)	NIRS (4 Hz) – 24 source-detector measurement, no specific equipment information provided<br><br><u>Optical density converted to HbO and HHb</u>	Pre-frontal cortex<br><br>Children started at resting and then watched movie	Algorithm is adjusted autoregressive model with pre-whitening filter and robust regression to decrease effects of physiological and artifact based noise (AR(P)-IRLS)<br><br>Evaluated based on AUC-ROC with p=0.05<br><br>AR(P)-IRLS had best performance in all artifacts (synthetic spike, synthetic shift, experimental)	Ordinary least squares (OLS) with and without pre-whitening<br><br>Wavelet based with OLS (wavelet OLS)<br><br>Spline and OLS (spline-OLS)	AR(P)-IRLS outperformed spline and wavelet methods in motion artifact correction<br><br>Pre-whitening decrease amount of false positives<br><br>The AUC-ROC values for the AR(P)-IRLS were consistently higher than all other compared metrics. The AUC value for the block task was consistently over 0.015 and for the event task was consistently over 0.025.	Proposed algorithm was able to remove both types of artifacts (physiological and motion-based) compared to ordinary least squared algorithm and other methods	4 Hz sampling rate inflated false positive errors, may have lead to worse results for certain methods evaluated<br><br>Method may work better if shift artifacts are removed during pre-processing
Barker et al. (2016) [34]	9 subjects (25 to 47 years old)	NIRS (10 Hz) – Techen CW6<br><br><u>HbO and HHb</u>	Frontal cortex<br><br>Subjects performing choice reaction time tasks with rests	Applies a Kalman estimator to the AR-IRLS model using a dual-stage Kalman filter such that it is able to be applied in real-time	Offline AR-OLS and AR-IRLS [60]	There was not much quanitative data provided regarding the successfulness of Kalman AR-IRLS compared to the offline AR-IRLS and AR-OLS.<br><br>Based on simulation results when 5 minutes of data is considered, the CNR of the signal was 0.5, 1, and 2, the Kalman model performed the best in sensitivity, was	Methods appears to adjust the signal in the presence of artifacts which are spikes in data	Do not directly identify artifacts, and only adjust signals to fit simulated model

						only slightly worse than the AR-IRLS in specificity and false positive rate		
Cui et al. (2010) [24]	10 healthy volunteers (mean age of 26.9)	NIRS (10 Hz) – Hitachi ETG-4000<br><br><u>HbO and HHb</u>	Bilateral motor cortex<br><br>Small head motions, finger tapping	CBSI		Spikes removed in Hb and HbO signals<br><br>CNR ratio to 2.59 from 1.31 and 1.28 before<br><br>Improves spatial specificity of signal	CSBI is simple to apply, can be fully automated, and can be applied offline or online<br><br>Improves signal quality, does not degrade signal when large amounts of noise present<br><br>Improves CNR, spatial specificity, and removes spikes	Conducted under ideal conditions, no indication for performance in clinical evaluation<br><br>No testing against other methods<br><br>Assumed that Hb and HbO are perfectly negatively correlated
Fishburn et al. (2019) [33]	23 healthy volunteers ages 7 to 15 years	NIRS (50 Hz, resampled at 5 Hz) – TechEn CW6 (NIRSOptix)<br><br><u>HbO and HHb</u>	Optodes placed in phantom on scalp<br><br>Subjects had to press buttons in certain order with fingers (working load tasks)	Temporal Derivative Distribution Repair (TDDR): compute temporal derivative of signal, initialize vector of observation weights, iteratively estimate the robust observation weights, apply resulting robust weights to centered temporal derivative to produce corrected derivative, integrate the corrected temporal derivative to yield corrected signal	CBSI [24]<br><br>MARA [41]<br><br>tPCA [28]<br><br>Kurtosis wavelet [23]<br><br>Spline Savitzky Golay filtering (Spline-SG) [32]	The rankings of the magnitude of AUC-ROC values were compared to the motion free data (0.869) were: TDDR (0.775), CSBI (0.733), spline-SG (0.652), tPCA (0.591), MARA (0.563), uncorrected data (0.516), and kWavelet (0.513).<br><br>The success of the artifact removal for the experimental data was evaluated using maximum activation t-statistic and greatest number of mesh vertices with positive significant ($p<0.05$) values. The ranking of the magnitude for t-statistic was: TDDR (4.88), kWavelet	TDDR performed better with pre-filtering (especially as sampling rate increased)<br><br>TDDR effective for both spikes and baseline shifts<br><br>Validated using simulated and empirical data	Performance dependant on sampling rate (high frequency components decrease effectiveness)<br><br>Low amplitudes and slow artifacts are not always removed

						(3.96), tPCA (3.79), spline-SG (3.68), uncorrected (3.67), CSBI (3.02), and MARA (2.96). The ranking of the magnitude for mesh vertices with positive significant values was: TDDR (2399), uncorrected (1560), spline-SG (1153), kWavelet (935), MARA (924), CBSI (903), and tPCA (891)		
Wang and Seghouane et al. (2019) [68]	Number of subjects not given<br><br>3 to 12 years of age	fNIRS (12.5 Hz) – NIRScout (NIRx)<br><br>HbO (Artificial and randomized artifacts introduced to signals)	N/A<br><br>Recorded at rest	Discrete cosine transformation coefficients were used to estimate the signal<br><br>Two parameters are basis function and robust parameter estimation	TARA (convex and non-convex) [74]	MSE between true resting state signal and restructured signal were 0.020876 for TARA, 0.015514 for TARA (Non-Convex) and 0.0037094 for the proposed method<br><br>The proposed method also had the best performance under varying signal interference conditions	Superior performance of proposed artifact compared to TARA and TARA (non-convex) by mean squared errors (MSE) and signal to interference ratio (SIR)	Synthetic artifacts<br><br>Need to further refine parameters for proposed algorithm
Raggam et al. (2020) [69]	14 healthy volunteers (5 female, 9 male with mean age 25 ± 3 years)	NIRS (3.91 Hz) – NIRStar (NIRx)<br><br>HbO and HHb<br><br>*ECG*<br>*BP*<br>*HR*<br>*respiratory frequency*	Optodes covering scalp<br><br>Performing hand exercises and motor imagery	Three methods in toolbox for reducing physiological artifacts in fNIRS<br><br>Common average reference (CAR) – systematic influences interfere with the signal in all channels and can be reduced by using the mean of all channels. Transfer function models – model to remove the perturbations of the physiological artifacts	N/A	No quantification	Indicated that physiological artifacts in fNIRS were successfully corrected	No quantification of results for artifact correction<br><br>NICA is only compatible with NIRScout 1624 device

				from HbO and Hb signals<br><br>Further artifact removal from physiological or technical sources accomplished with low bass Butterworth filter<br>Grand Average and Region of Interest Analysis – grand average across all subjects in each channel, and region of interest combines Hb and HbO data				
Sutoko et al. (2018) [70]	38 attention deficit/hyper activity disorder Children, with IQ over 70	fNIRS (0.8 Hz)<br><br>Previously collected data, signal type not specified	Forehead<br><br>Subject with and without treatment went through tasks	Algorithm based on 3 methods sudden increase, shifted baseline, and discrepancy of correlation	visual-based rejection	71.8% rejection accuracy	Propose a route to automated artifact detection	Limited accuracy overall, method is overall insufficient to address all artifact issues
Robertson et al. (2010) [17]	3 subjects, demographic information not specified	NIRS (1.8 Hz) – NIRX DYNOT<br><br>Used wavelengths corresponding to HbO and HHb, but did not state	Left and right motor cortices<br><br>Shake, tilt, and nod head (3 subjects), finger tapping (1 subject)	Two-input Recursive Least Squares Adaptive Filter<br><br>Discrete wavelet (symlet 8 wavelet) transformation using thresholding of coefficients<br><br>**Two channel multiple regression**	Each methodology presented in article was compared to each other	When time of motion was known (set of 3 subjects) the average across the three subjects for SNR (dB):<br>For λ=760nm, Wavelet (5.95), 30-channel regression (5.67) and ICA (5.62) had the best performance. For λ=830nm, the ranking was the same but Wavelet (4.93) and 30-channel regression (4.56) performed dramatically	ICA and 30-channel regression performed the best<br><br>Wavelet performs well when the location of the motion is known	Multi-channel regression and ICA requires the use of several channels<br><br>Without known motion, the SNR when wavelet is used drops dramatically

				**Multi-channel (30) regression**<br><br>ICA-based method		better than ICA (2.75) in this occasion.<br><br>However, when the motion was not known: ICA (λ = 760nm – SNR = 3.20 dB; λ = 830nm SNR = 3.67 dB) and 30-channel regression (λ = 760nm – SNR = 3.01 dB; λ = 830nm – SNR = 2.54 dB) outperformed wavelet (λ = 760nm – SNR = 0.89 dB; λ = 830nm – SNR = 0.58 dB).<br><br>The RLS method and 2-channel regression method had substantially worse performance than the rest.		Small cohort
Sweeney et al. (2013) [35]	10 healthy subjects, 6F/4M (29 ± 5.6 years)	Refer to previous work by this author [31]<br><br>*EEG recorded but not used as auxiliary signal*	Refer to previous work by this author [31]	Ensemble empirical mode decomposition with canonical correlation analysis (EEMD-CCA) which decomposes signals into multidimensional signal using EMD and isolates artifacts using CCA second-order statistics	Ensemble empirical mode decomposition with independent component analysis (EEMD-ICA) [71]<br><br>Wavelet-based method [17]	Wavelet method had ΔSNR of 3.1 dB, 43.6% artifact reduction, and 0.66 correlation with ground truth signal<br><br>EEMD-ICA method had ΔSNR of 3.4 dB, 43.4% artifact reduction, and 0.66 correlation with ground truth signal<br><br>EEMD-CCA method had ΔSNR of 3.5 dB, 49.4% artifact reduction, and 0.68 correlation with ground truth signal	EEMD-CCA method outperforms the other two methods by all metrics.	No significant computational differences using CCA

Where AR(P)-IRLS = Adjusted Autoregressive Model with Pre-whitening Filter and Iteratively Reweighted Least Squares, AUC-ROC=Area Under Receiver Operating Characteristic Curve, BP = Blood Pressure, CNR = Contrast-to-Noise Ratio, CSBI = Correlation-Based Signal Improvement, ECG = Electrocardiography, EEG = Electroencephalography, fMRI = Functional Magnetic Resonance Imaging, fNIRS = Functional Near-Infrared

Spectroscopy, HHb= Concentration of Deoxyhemoglobin, HbO= Concentration of Oxyhemoglobin, HR = Heart Rate, MARA = Movement Artifact Reduction Algorithm, MSE = Mean Squared Error, N/A = Not Applicable, NIRS = Near-Infrared Spectroscopy, OLS = Ordinary Least Squares, PRD = Percent Root Difference, R = Pearson's Correlation Coefficient, RMSE = Root Mean Squared Error, SG = Savitzky-Golay Filtering, SNR = Signal-to-Noise Ratio, TARA = Targeted Artifact Removal Algorithm, TDDR = Temporal Derivative Distribution Repair, tPCA = Targeted Principle Component Analysis. **Bold text indicates that there were several methods that were presented in the article, bold indicates the highlighted method.**

Table S8. Signal quality improvement and physiological/other noise removal methods - Signal drift removal methods.

Reference	Subject Information	Data Type (Sampling Rate) – System Used<br><br><u>Signal measured</u><br><br>*Auxiliary signals measured*	Sensor Location and Instruction to Subjects Signal Recording	Artifact Removal Method(s) Used	Methods Compared	Effectiveness	Study Results and Conclusions	Limitations
Shah and Seghouane (2014) [25]	10 healthy volunteers (4 male) with mean age of 26.9	fNIRS (10 Hz) – Hitachi ETG-4000<br><br><u>HHb and HbO</u>	Left motor cortex<br><br>Performed tasks involving finger tapping and head motion	Based on a consistent estimate of the HRF, drift is estimated using a wavelet thresholding method applied to the residuals generated by removing the estimated induced functional response from the fNIRS times series	Wavelet-MDL method [61]	Proposed drift estimating method had CNR above 6 for all HbO channels and above 5 for all HHb channels (closest to ground truth signal). Wavelet-MDL method had values for CNR in both HbO and HHb below 3 for all channels	Superior performance to other de-drifting methods	Only removes drifting
Seghouane and Ferrari (2019) [78]	2 patients Shah and Seghouane [25]	Presented by Shah and Seghouane [25]	Presented by Shah and Seghouane [25]	HRF estimation procedure to minimize the impact of unexpected noise using method presented by Shah and Seghouane [25]	Two methods presented by Ye et al. in a NIRS SPM toolbox [79]	Indicates superior performance in estimating HRF when noise and drift are present	Results indicate superior performance in comparison to NIRS SPM toolbox methods	Looks at trend errors of the data, and requires high frequency sampling<br><br>Insufficient quantification of results

Where CNR=Contrast-to-Noise Ratio, fNIRS=Functional Near-Infrared Spectroscopy, HHb= Concentration of Deoxyhemoglobin, HbO= Concentration of Oxyhemoglobin, HRF= Hemodynamic Response Function, MDL=Minimum Description Length, SPM= Statistical Parametric Mapping

Table S9. Signal quality improvement and physiological/other noise removal methods - Physiological and other noise artifact removal methods – NIRS only.

Reference	Subject Information	Data Type (Sampling Rate) – System Used<br><br><u>Signal Measured</u><br><br>*Auxiliary Signals Measured*	Sensor Location and Instruction to Subjects Signal Recording	Artifact Removal Method(s) Used	Methods Compared	Effectiveness	Study Results and Conclusions	Limitations
Nguyen et al. (2018) [80]	5 healthy male volunteers (mean age of 36.2, 33-37)	NIRS (1.81 Hz) - Dual-wavelength continuous-wave fNIRS (DYNOT, NIRx)<br><br><u>HbO and HHb</u>	Left motor cortex<br><br>Thumb and little movements	RLSE method with linear model of known frequencies of physiological occurrences to correct signal	Kalman filter<br><br>Low-pass filter<br><br>ICA	Noise reduced in HbO and HHb reduced by 77 and 99%, respectively by CNR.<br><br>Proposed method gives extracted heart rate more consistently than Kalman filter, low-pass filter, and ICA<br><br>T-value greater for proposed method in 4 of 5 subjects compared to Kalman and LPF	Compared to Kalman filter and low-pass filter, it performed better and could extract HR signal<br><br>Proposed model can be used for noise removal and HR extraction both online and offline	Larger cohort needed<br><br>Frequencies of physiological noises were assumed constant
Zhang et al. (2007) [72]	1 healthy 36 year old male volunteer	NIRS (200 Hz) – TechEn NIRS1<br><br><u>HbO and HHb</u>	Primary/secondary visual cortex<br><br>Visual stimulation task	Low pass 1.25 Hz sixth order Butterworth filter<br><br>Bandpass filter to remove any drift<br><br>Monte Carlo simulation with common head structure and tissue optical properties, differential path length factors (DPF) chosen	Before and after filtering	Adaptive filtering reduced the physiological interference due to low-frequency oscillations (10 db less)<br><br>CNR analysis in certain bands of HbO data indicate CNR going from 40.2% to 80.8%<br><br>In HHb, power spectral density suggests that CNR is decreased by 23.5%	Indicates that in HbO, adaptive filter works well unlike in HHb, this is due to the lack of dominance of global interference in HHb signals	Poor performance in HHb signals<br><br>Only single subject

				HbO concentration changes from source-detector pairs fed into adaptive filter<br><br>Adaptive filter using finite impulse response (FIR) and transversal structure (tapped delay line) with 100 nodes, and Windrow-Hoff Least Mean squared adaptation algorithm				
Zhang et al. (2009) [81]	17 healthy volunteer (10 male and 7 female, mean age of 20.6 years)<br><br>2 not included due to technical issues	NIRS (200 Hz) – TechEn NIRS1<br><br><u>HbO and HHb</u>	Primary/secondary visual cortex<br><br>Exposed to visual stimuli	Data offset-corrected and digitally low-pass filtered at 5 Hz<br><br>Band pass filtered between 0.01 Hz to 1.25 Hz (remove signal drift and other noise)<br><br>Adaptive filter for HbO and HHb filtered separately using finite impulse response (FIR) and transversal structure (tapped delay line) with 100 nodes, and Windrow-Hoff Least Mean squared adaptation algorithm	Before and after filtering	Of HbO measurements 71% show CNR increase (r>0.6)<br><br>All measurements (156), 49% show some CNR increase<br><br>Average CNR for HbO before and after are 64% and 75%, respectively<br><br>For HHb, average CNR 85% and 63% before and after adaptive filtering, respectively (decrease in effectiveness)	Functions better when r>0.6<br><br>Functions better when SNR is higher	Method not very effective for HHb, decrease in CNR

Ortega-Martinez et al. (2022) [29]	Two data sets<br><br>Set #1 - 14 healthy volunteers<br><br>Set #2 - 10 healthy volunteers	NIRS – TechEn CW6 (NIRSOptix)<br><br>Set #1 – sampling rate not given<br><br>Set #2 – 100 Hz<br><br><u>HbO and HHb</u>	Set #1 – visual cortex and at rest<br><br>Set #2 – motor cortex and finger tapping	Steps in online algorithm include: low pass filter, Beer-Lambert law, mean average high-pass filter, tCCA calculation, Kalman filter<br><br>Kalman filter tuned using first data set synthetic data<br><br>Set #1 - used to tune strategies with synthetic HRF<br><br>Set #2 - used to demonstrate online processing	General Linear Model	Kalman filter tuning improves RMSE over GLM compared to no tuning (60% smaller for Kalman filter tuning using proposed method compared to GLM)	Selective reduction in physiological noise as well as reduced high frequency noise<br><br>Resulted in higher contrast to noise ratio<br><br>Determine of if hemodynamic response function originates from left or right finger tapping is good with proposed classification method	With different types of stimuli, the HRF components must be reset
Santosa et al. (2013) [82]	8 healthy volunteers (males, age from 23 to 33)	NIRS (1.81 Hz) – DYNOT (NIRx)<br><br><u>HbO and HHb</u>	Prefrontal cortex<br><br>Asked to do arithmetic tasks	ICA	Low-pass filtering	Post-ICA had higher t-values, SNR for HbO improved from 0.66 to 4.33	ICs obtained corresponded with low-frequency noise, respiratory noise, and other noise<br><br>Could effectively separate motion artifacts	Small cohort<br><br>Not clear how motion artifacts are removed (which IC)
Chi et al. (2018) [83]	No information about subjects	NIRS – 'NIRS signal' – no equipment information provided<br><br><u>No information about sampling rate</u>	N/A	Empirical mode decomposition<br><br>ICA<br>Correntropy Spectral Density	Measured data without noise	Heart rate estimate is 80-90% accurate	Heart rate measured using this method is no different than if measured without noise	Not validated on any other noises/artifact types. No information about data set used

Santosa et al. (2020) [84]	12 right-handed healthy volunteers (5 male, 7 female) age range 20 to 50 years	NIRS (7.8125 Hz) – NIRScout (NIRx)<br><br><u>HbO and HHb</u>	Covering scalp over motor cortex Lights off in room, imagery prompts (ex. walking), different stimuli, and breath work	Purpose was to compare different pre-processing and data processing techniques for subjects under different brain stimuli<br><br>Preprocessing include:<br>None<br>bPCA<br>PCA<br>SS filter<br><br>Data processing include:<br>OLS<br>AR-IRLS<br>OLS with SS regression<br>AR-IRLS with SS regression<br>Mixed-effect AR-IRLS with SS regression	N/A	AR-IRLS had best performance for type-I errors in all three states of data tested (FPR to 40% from 60%)<br><br>Best performance in AUC-ROC across data sets was SS regression + ME + AR-IRLS method; close behind was SS regression + AR-IRLS method, pre-processing none, PCA, and bPCA did not have dramatic effect on AUC-ROC	AR-IRLS regression model had better control of false positive error compared to OLS methods, ME was slightly better but higher computational cost<br><br>AR-IRLS performed well in RS data<br><br>Better performance using short separation filter and short separation in regression	
Guerrero-Mosquera (2016) [85]	17 no history of neurological or psychiatric disorders were recruited for this study (7 male, 4 left-handed; group mean age 26.93 ± 4.65 years	16 NIRS channels (10Hz) - BrainSight<br><br><u>HbO and HHb</u>	Frontal, dorsolateral prefrontal, parietal and occipital areas<br><br>12 experimental blocks (2-back and 0-back responses)	Relies upon the assumed negative correlation between HbO and HHb. Running correlation (global information) of the signal obtained from sliding windows	Cross correlation coefficient for local correlations	Under different cognitive conditions (2-back and 0-back tasks) the AUC-ROC was 60.57% using running correlation (global correlations) and 91.23% using cross correlations (local correlations)	Technique for the automatic detection of noisy channels in the recording of multi-channel fNIRS signals.<br><br>Global correlations are insufficient to detect noisy channels	Limited success of global correlations<br><br>Computational cost for long fNIRS signals

Where AUC-ROC=Area Under Receiver Operating Characteristic Curve, AR-OLS = Iterative Autoregressive Ordinary Least Squares, AR-IRLS= Iterative Autoregressive Least Squares, bPCA = Baseline Principle Component Analysis, CNR = Contrast-to-Noise Ratio, DPF = Differential Path Length Factors, FIR = Finite Impulse Response, fNIRS= Functional Near-Infrared Spectroscopy, GLM = Generalized Linear Model, HHb= Concentration of

Deoxyhemoglobin, HbO= Concentration of Oxyhemoglobin, HR = Heart Rate, HRF = Hemodynamic Response Function, IC = Independent Component, ICA = Independent Component Analysis, LPF = Low-Pass Filter, N/A = Not Applicable, ME = Mixed Effect Version, NIRS = Near-Infrared Spectroscopy, OLS = Ordinary Least Squares, PCA = Principle Component Analysis, SNR = Signal-to-Noise Ratio, SS = Short-Separation, tCCA = Targeted Canonical Component Analysis

Table S10. Signal quality improvement and physiological/other noise removal methods - Physiological and other noise artifact removal methods – Auxiliary signals.

Reference	Subject Information	Data Type (sampling Rate) – System Used<br><br>Signal Measured<br><br>*Auxiliary Signals Measured*	Sensor Location and Instruction to Subjects Signal Recording	Artifact Removal Method(s) Used	Methods Compared	Effectiveness	Study Results and Conclusions	Limitations
Bauern-field et al. (2014) [26]	12 healthy volunteer (7 female and 5 male) with average age of 22.5 (22-27)	NIRS (10 Hz) – Hitachi ETG-4000<br><br>HbO and HHb<br><br>*BP*<br>*ECG*<br>*Respiration*	Above sensorimotor cortex<br><br>Motor functions conducted	Signal processing approaches: CAR method, TF models, and ICA are applied	Based changes in squared coherence between IC and BP and respiratory signals based on power spectral density	Improvement of CNR in deoxy Hb using TF, no improvement using CAR or ICA<br><br>The CAR approach the subject specific CNR improvements for HbO ranged from −16.0% to 223.4% and for HHb from −28.6% to 19.0%<br><br>For TF, CNR improvements for HbO was in the range from 3.7% to 188.8% and for HHb from −0.6% to 55.0%<br><br>For ICA, HbO signals improved between −23.9% and 33.0% (median: 5.3%, MAD: 8.7), and for HHb signals demonstrated CNR changes from −51.7% to −9.3%<br>CAR, ICA, and TF produced reductions in respiratory and BP waves	Methods are promising in reduction of global influences from HbO using CAR, ICA, and TF<br><br>TF promising in global influence reduction in deoxy Hb	Results are not very clear in presentation

						Improvement in HbO using ICA, TF, and CAR		
Kohno et al. (2007) [87]	6 right-handed healthy volunteer males (37 to 52)	NIRS (sampling rate not given) – no equipment information provided<br><br>HbO<br><br>*Skin blood flow using transcranial Doppler*	12 sources and 12 sensors on forehead<br><br>Rest, locomotor task then rest again	Independent component analysis (MS-ICA) (ICA algorithm proposed by Molgedey and Schuster [90])	N/A	The coefficient of correlation was 0.724 between identified component and changes in the skin blood flow for first patient and coefficient of correlation was 0.789 for second patient	MS-ICA is useful for skin blood flow artifact elimination	Small cohort
Sato et al. (2016) [88]	15 healthy volunteers (22-67 years, seven men and eight women)<br><br>1 stroke patient (60 years old, 6 years after stroke)	fNIRS (no sampling rate given) – FOIRE-3000 (Shimadzu Corp)<br><br>HbO and HHb<br><br>*fMRI*	Optodes covering both motor hemispheres of the brain<br><br>Performed tapping with finger or grasping with hand	Three-tiered artifact reduction method includes: preprocessing, estimation of global scalp-hemodynamic artifacts, and removal of scalp-hemodynamics using GLM analysis	Standard GLM (RAW)<br><br>MS-ICA method<br><br>(ICA algorithm proposed by Molgedey and Schuster, [90])<br><br>RestEV method (an eigenvector-based spatial filtering method using rest proposed [75]	ShortPCA GLM method had an adjusted R^2 value much higher than other methods<br><br>ShortPCA had the highest specificity (over 0.8), RAW had highest sensitivity with fMRI comparison (over 0.8) in uncorrelated groups.<br><br>In corelated groups, ShortPCA has highest specificity (near 0.5), all methods have close to 0.8 in specificity	ShortPCA GLM method is most appropriate for fitting changes in oxyhemoglobin during movements	No improvement to deoxyhemoglobin<br><br>Needs to be validated for different optode lengths
Von Lühmann et al. (2020) [30]	14 healthy volunteers (21 years mean age, 11 male/3 female)	fNIRS (50 Hz) – TechEn CW6 (NIRSOptix)<br><br>HbO and HHb<br><br>*Blood pressure PPG Accelerometer*	Occipital lobe<br><br>N/A	GLM with tCCA and modelling of physiological nuisance regressors	GLM with short separation (SS)	Superior performance of GLM with tCCA compared to GLM with SS in simulated data. For HbO at the smallest CNR resulted in an increase of 45% in correlation, a decrease of 55% in RMSE and 3.25 times increase in F-score.	New method increases robustness of HRF estimation without adding computational load	

						In real data, more significant channels in HHb and HbO using GLM with tCCA (6.9 in HbO and 5.2 in HHb) compared to GLM with SS (4.2 in HbO and 3.8 in HHb), lower p-value for GLM with tCCA for HbO (0.03) compared to GLM with SS (0.14), not very different for HHb (0.08 for GLM with tCCA and 0.14 for GLM with SS)		
Bontrager et al. (2014) [86]	7 healthy volunteers, no demographic information provided	fNIRS (50 Hz) – Adult flexible sensor (ISS Inc.)<br><br>NIRS signal, likely HbO and HHb<br><br>*BP respiration*	Left premotor and primary motor cortex<br><br>Lying in a supine position conducting a pinching task	Adaptive filter updated based on mutual information extracted using machine learning such that physiological noise is minimized in fNIRS data	Recursive Least Squares (RLS)<br><br>Raw data	Filter performance evaluated for simulated data with blood pressure, simulated data with HRF, and real data with BP; mutual information filter had largest reduction in mutual information, RLS had slightly smaller cross-correlation function values<br><br>For Simulated data-BP: mutual information was 0.39 for raw, 0.31 for RLS filtered, 0.12 for MI-filtered<br><br>For Simulated data-HRF: mutual information was 0.51 for raw, 0.48 for RLS filtered, 0.76 for MI-filtered<br><br>For real data-BP: mutual information was 0.21 for raw, 0.10 for RLS-filtered, 0.06 for MI-filtered	MI filter more successful at reducing correlations between fNIRS and BP	Worse performance when simulated data compared to HRF

Where BP = Blood Pressure, CAR = Common Average Reference, ECG = Electrocardiogram, GLM = Generalized Linear Model, HHb= Concentration of Deoxyhemoglobin, HbO= Concentration of Oxyhemoglobin, HRF = Hemodynamic Response Function, ICA = Independent Component Analysis, MI = Mutual Information, NIRS = Near-Infrared Spectroscopy, PPG = Photoplethysmography, R^2 = Coefficient of Determination, RLS = Recursive Least Squares, SS = Short Separation, TF = Transfer Function, tCCA = Targeted Canonical Component Analysis
